# The association between comorbidities and stigma among breast cancer survivors

**DOI:** 10.1038/s41598-022-15460-8

**Published:** 2022-08-11

**Authors:** Yuxin Zhang, Jie Zhao, Nan Jiang, Yongyi Liu, Ting Wang, Xi Yu, Jiwei Wang, Jinming Yu

**Affiliations:** 1 School of Public Health, Key Laboratory of Public Health Safety of the Ministry of Education and National Health Commission Key Laboratory of Health Technology Assessment, Fudan University, Shanghai, 200032 China; 2 School of Public Health, University of Washington, Seattle, WA 98195 US; 3 Qingpu District Center for Disease Control and Prevention, Shanghai, 201799 China

**Keywords:** Breast cancer, Health care

## Abstract

This study aimed to explore the association between types and numbers of comorbidities and stigma among breast cancer survivors (BCSs). A cross-sectional study was conducted among 937 BCSs in Shanghai Cancer Rehabilitation Club. All participants were asked to fill in an online questionnaire including Stigma Scale for Chronic Illnesses 8-item version (SSCI-8) and questions on sociodemographic characteristics and health status. Multivariate linear regression was used to analyze the association between comorbidities and stigma, adjusting for confounding factors. Results showed that nearly 70% of the participants had one or more comorbidities. The participants with stroke, digestive diseases or musculoskeletal diseases had significantly higher stigma than those without the above comorbidities. In addition, stigma was higher among survivors in the group with a greater number of comorbidities. Thus, it is important to strengthen the management of stigma in BCSs, especially for those with comorbidities.

## Introduction

*Global cancer statistics 2020* shows that breast cancer has ranked first in the incidence of malignant tumors in women worldwide^1^. Meanwhile, with the advancement of medical technology and effective breast cancer screening and treatment, more and more breast cancer patients are surviving for a long time^2^. The diagnosis and treatment have long-term physical and psychological effects on these breast cancer survivors (BCSs)^3^.

Stigma is a negative psychological experience caused by a certain disease^4^, and it is one of the psychological problems that BCSs often face^5^. It refers to a negative social perception and a sense of shame or stigmatization caused by negative remarks from some certain people^6^. Corrigan and colleagues distinguished stigma in felt/perceived stigma (the awareness of the discriminatory stereotype around the own illness), enacted stigma (being discriminated against or treated unfairly by others in actual encounters), and internalized/self stigma (internalization and acceptance of the discriminatory stereotype)^7,8^. BCSs inevitably face the decline of physical function and severe psychological pressure, and encounter difficulties in work and interpersonal communication^5,9^. Coupled with the high cost of treatment and care, cancer survivors often feel that they are a serious burden to their families, resulting in self-denial and stigma^10^. When stigma is internalized, the negative perception can lead to negative emotions and attitudes such as shame, guilt, depression, fear of discrimination and the disease itself^11–13^. Stigma of BCSs may also be related to some cultural factors. Chinese culture is greatly influenced by Buddhist thought, which promotes karma and retribution^14^. Under the influence of this Buddhist ideology, people believe that cancer is the result of karma. Therefore, people are prone to blame disease on their own fault and develop a sense of stigma. This deep-rooted perception causes cancer survivors to be questioned and discriminated against at work and in interpersonal interactions, bringing about self-denial and stigma, which in turn has a negative impact on behavior and health. A long-term sense of shame can lead to constant feelings of inferiority and social exclusion, and even cause serious psychological stress^15^. Withdrawal and avoidance of shame usually lead to stress, anxiety or depression^16,17^, and at the same time bring problems such as poor treatment compliance and delayed medical treatment, which eventually have a negative impact on the quality of life (QOL) of patients^18^.

Studies with large samples have found higher levels of stigma in breast cancer patients than in patients with other gynecological diseases^19^. Most BCSs will undergo surgery, chemotherapy and other treatments. The nature of BCSs’ survival and the aftermath of the disease that perhaps left physical scarring or removal that are visible and therefore make survivors more prone to be victims of stigma^10,20^. At the same time, BCSs generally report more comorbidities than patients without a history of cancer^21–23^, and the burden of comorbidity is becoming one of the main health problems of these patients^24–26^. Studies have shown that breast cancer combined with comorbidity is associated with poorer health-related QOL^27^. For BCSs, the management of coexisting chronic diseases and their attendant burdens is also an important issue in long-term survival^28^.

Stigma is also present in other diseases such as Acquired Immune Deficiency Syndrome (AIDS), mental illness, cancers, stroke, multiple sclerosis and epilepsy^4,6–8,29–31^. A study showed that the overall level of internalized stigma of patients with anxiety disorders (n = 109) was positively associated with a comorbid personality disorder^32^, and the explanation may relate to the finding that individuals with comorbid personality disorder often experienced more severe symptoms of anxiety and depression and thus elevated the level of internalized stigma^32,33^. Another study found that felt stigma of individuals with HIV (n = 201) was associated with poor overall health, comorbid depression, anxiety and alcohol dependence^34^. Besides, a study showed that perceived alcohol stigma was significantly higher in those with internalizing comorbidity as compared to those with no comorbidity or externalizing comorbidity^35^. As yet, few studies have examined whether breast cancer combined with comorbidity is associated with more severe stigma.

This study aimed to investigate associations between comorbidities and stigma among BCSs. We hypothesized that: (1) BCSs with a particular type of comorbidity had an increased stigma; (2) the stigma was higher among BCSs with more comorbidities. Clarifying these associations may be helpful to identify priority BCS populations that may be at high risk for stigma, and it has practical implications for providing ideas for stigma intervention practices in Chinese BCS populations.

## Methods

### Setting and participants

In this cross-sectional study, all the participants were recruited from registered members of Shanghai Cancer Rehabilitation Club (SCRC) using a convenience sampling method^36^. SCRC is a non-government organization that has a three-level management network of the city, district, and street^37^. It recruits members from communities and hospitals through extensive recruitment channels covering all sixteen districts of Shanghai^36^. Each street or town has a WeChat group that includes all the members of that street who have registered in SCRC^36^. There is a total of about 50,000 BCSs in Shanghai, and about 5,000 registered BCSs in SCRC^37^. Before the participants were recruited, we estimated the sample size required, using the sample size formula $\mathrm{N}={(\frac{{\mathrm{z}}_{1-\propto /2}\upsigma }{\updelta })}^{2}$ for the cross-sectional study. In an Iranian study of 233 female breast cancer survivors recruited at three cancer centers, the SSCI-8 scale was used to assess the level of stigma of the participants^38^. The average stigma score was obtained by reviewing the literature as 1.47 with a standard deviation σ of 0.19^38^. δ was taken as 0.01 mean score and α as 0.05. A final sample size N of at least 642 was required. Recruitment invitations were sent to sixteen district-level SCRCs^39^. From March to April 2021, four districts were recruited on a first-come, first-served basis. Finally, all BCSs registered with the SCRC in these four districts, a total of 1496 BCSs in 41 WeChat groups (one for each street or town), were recruited^39^. The questionnaire was sent to each participant via the WeChat group, which means the data of this study was collected through an online survey^39^. All BCSs in SCRC could access recruitment information through the WeChat groups^39^. The inclusion criteria for this study included: (1) female and above 18 years old; (2) breast cancer as the first primary cancer and willing to complete treatment; (3) be able to participate in cancer club activities independently; (4) no cognitive impairment^36^. Participants who met the inclusion criteria were included in the study. Finally, 1012 questionnaires were returned. The response rate was 67.65%. Among them, 937 questionnaires were valid and the valid rate was 92.59%.

### Instruments

Data were collected using a self-reported structured questionnaire that included questions about sociodemographic characteristics (age, marital status, education level and income), health status (body mass index [BMI], comorbidity, surgery, radiotherapy, chemotherapy, and other treatment methods, recurrence, metastasis, and time since diagnosis), and stigma.

### Comorbidities

Participants were asked to answer either “yes” or “no” on a list of physician-diagnosed chronic diseases including diabetes mellitus, heart and cardiovascular diseases, stroke, respiratory diseases (e.g., asthma, chronic bronchitis, and chronic obstructive pulmonary disease), digestive diseases (e.g., fatty liver, chronic hepatitis, peptic ulcer, gallbladder polyp, gallstone, and hemorrhoids), and musculoskeletal diseases (e.g., osteoarthritis and rheumatoid arthritis)^40^. These chronic diseases must be clinically diagnosed by secondary or tertiary hospitals in China.

### Stigma

Stigma was measured using the Stigma Scale for Chronic Illnesses 8-item version (SSCI-8), which evaluates stigmatization as a psychosocial concept referring to any act, thought, attitude, or perception toward a person with chronic conditions^36,41^. SSCI-8 is an 8-item newly developed short-form instrument, which is appropriate for patients with chronic illnesses^36^. The items are scored based on a five-point Likert scale from “never” to “always”; patients respond to items based on their personal experiences, whether they are subjected to any enacted stigma or felt stigma internally^36^. In this study, average score of the scale (ranging from 1 to 5 points) was used to evaluate stigma of the participants. The higher the score, the higher the level of stigma. The SSCI-8 has been proved to have good reliability and validity in a variety of chronic diseases^36,41,42^. However, it has not yet been used in the Chinese breast cancer population^36^. Therefore, after obtaining permission to translate and use the questionnaire, the research team translated the questionnaire, following these steps: (1) Two native Chinese-speaking experts independently translated the original SSCI into simplified Chinese. A third native Chinese-speaking expert reconciled their translations and combined a hybrid version as the initial Chinese version. (2) The initial Chinese version was back-translated into English by a native English-speaking expert who was unaware of the original English version of the questionnaire. (3) Three more experts (native Chinese speakers) were eventually invited to check the translated and back-translated versions to select the most appropriate translation for each item or provided alternate translations to fit the Chinese context if the provided translations were unacceptable. (4) The Chinese version was considered final when there were no substantial differences^36,39^. A final quality review by the project members has performed again after the cognitive debriefing step was conducted, and the translation was finalized^36,39^. In this study, the Cronbach’s alpha and the structural validity coefficient of SSCI-8 were 0.900 and 0.909 respectively.

### Statistical analysis

Means and standard deviations were calculated for continuous variables (age, BMI, time since diagnosis), and numbers and percentages were computed for categorical variables (marital status, education level, household per capital income, surgery, radiotherapy, chemotherapy, endocrine drug therapy, hysterectomy, recurrence, metastasis). The differences in the distribution of stigma in different demographic characteristics were also analyzed using Kruskal–Wallis H test for multi-categorical variables and Mann Whitney U test for dichotomous variables. These variables were also included as confounders in later regression analyses for the association between types or numbers of comorbidities and stigma. In order to compare the level of stigma among BCSs in different age groups, different BMI and different time of diagnosis, we transformed age, BMI and time of diagnosis into classification variables (age: < 50, 50–59, 60–69, ≥ 70 years; BMI: < 18.5 kg/m^2^, 18.5–24.9 kg/m^2^, 25.0–29.9 kg/m^2^, ≥ 30 kg/m^2^; time of diagnosis: < 3 years, 3–5 years, 5–10 years, ≥ 10 years) during Kruskal–Wallis H test. However, they were still included as continuous variables in multiple linear regression models. The choice of sampling strategy probably had an effect on the non-normal distribution of the outcome variable. The data in the study did not conform to assumptions of normality and/or homoscedasticity/homogeneity of variance^36^. Research has shown that parametric tests (e.g., multiple regression, ANOVA) can be robust to modest violations of these assumptions^43^. Referring to the methods provided by other studies to address non-normality^44,45^, we performed data transformation—the stigma variable was inverted transformed. After transformation, each regression model included in this study met the assumption of normality and homoscedasticity/homogeneity of variances. Each regression model included one comorbidity or the number of comorbidities and the above-mentioned confounding variables. The multi-categorical variables (education level, household per capital income) were converted into dummy variables before being included in the regression models. The multi-categorical variable number of comorbidities was also converted to a dummy variable before being included in the regression model. All statistical analyses were performed by SPSS version 20.0. A two-sided *p* value less than 0.05 was considered as the significant level.

The study was carried out in accordance with relevant guidelines and regulations and approved by the Medical Research Ethics Committee of the School of Public Health, Fudan University (The international registry no. IRB00002408 and FWA00002399). Informed consent was obtained from each participant prior to the start of the survey.

## Results

### Basic demographic characteristics and treatment

Table 1 presents participant characteristics. The average age of participants in this study was 60.55 years (SD = 6.98). BCSs in this study were mainly aged 50 and above (92.4%), and 58.5% of the survivors were over 60 years old. Most BCSs were educated in high school (45.1%) and below high school (38.4%). 86.1% of them have been married. The survival duration of most BCSs (from the first diagnosis to the survey) was no less than 5 years (79.5%). Almost all participants had surgery (99.6%) or chemotherapy (89.1%); 42.9% and 66.9% had radiotherapy and endocrine drugs respectively; 12.3% had hysterectomy. The recurrence and metastasis rates were 5.3% and 5.7% respectively. Besides, the mean survival duration for participants in this study was 10.10 years (SD = 6.45).

**Table 1:** Average score of stigma in BCSs among various basic characteristics.

Characteristics	N (%) or mean (SD)	Mean (SD) of Stigma	Z/X^2^	*p*
**Age (years)**			1.320	0.724
**Mean (SD)**	60.55 (6.98)			
< 50	71 (7.6)	1.55 (0.065)		
50–59	318 (33.9)	1.63 (0.034)		
60–69	492 (52.5)	1.62 (0.026)		
≥ 70	56 (6.0)	1.57 (0.073)		
**Marital status**			− 1.651	0.099
Married	807 (86.1)	1.61 (0.021)		
Unmarried/widowed/divorced	130 (13.9)	1.69 (0.052)		
**Education**			4.180	0.124
< High school	360 (38.4)	1.63 (0.034)		
High school	423 (45.1)	1.58 (0.027)		
> High school	154 (16.4)	1.68 (0.045)		
**Household per capital income (yuan/month)**			1.881	0.390
< 4000	278 (29.7)	1.65 (0.037)		
4000-	389 (41.5)	1.58 (0.028)		
≥ 6000	270 (28.8)	1.64 (0.036)		
**BMI**			1.919	0.589
**Mean (SD)**	23.53 (2.93)			
Underweight (< 18.5 kg/m^2^)	19 (2.0)	1.72 (0.160)		
Normal weight (18.5–24.9 kg/m^2^)	614 (65.5)	1.63 (0.024)		
Pre-obesity (25.0–29.9 kg/m^2^)	218 (23.3)	1.59 (0.040)		
Obesity (≥ 30 kg/m^2^)	86 (9.2)	1.55 (0.060)		
**Treatment**				
**Surgery**			− 1.181	0.238
Yes	933 (99.6)	1.62 (0.019)		
No	4 (0.4)	1.34 (0.304)		
**Radiotherapy**			− 1.184	0.236
Yes	402 (42.9)	1.65 (0.031)		
No	535 (57.1)	1.59 (0.024)		
**Chemotherapy**			− 1.243	0.214
Yes	835 (89.1)	1.63 (0.020)		
No	102 (10.9)	1.56 (0.059)		
**Endocrine drug**			− 1.274	0.203
Yes	627 (66.9)	1.63 (0.023)		
No	310 (33.1)	1.59 (0.033)		
**Hysterectomy**			− 0.833	0.405
Yes	115 (12.3)	1.58 (0.055)		
No	822(87.7)	1.62 (0.021)		
**Recurrence**			− 0.954	0.340
Yes	50 (5.3)	1.72 (0.095)		
No	887 (94.7)	1.61 (0.020)		
**Metastasis**			− 2.251	0.024
Yes	53 (5.7)	1.79 (0.087)		
No	884 (94.3)	1.61 (0.020)		
**Time since diagnosis (years)**			4.360	0.225
Mean (SD)	10.10 (6.45)			
< 3	61 (6.5)	1.51 (0.078)		
3-	131 (14.0)	1.66 (0.054)		
5-	364 (38.8)	1.64 (0.032)		
≥ 10	381 (40.7)	1.60 (0.028)		

SD, standard deviation.

Table 1 also shows the distribution of stigma in different demographic characteristics and treatments. Survivors with metastasis (average score of stigma was 1.79) had higher stigma scores than those without metastasis (average score of stigma was 1.61), and the difference was statistically significant (*p* = 0.024). No statistically significant differences in stigma were found among other demographic characteristics or treatment.

### Comorbidities and stigma

**Figure 1:**
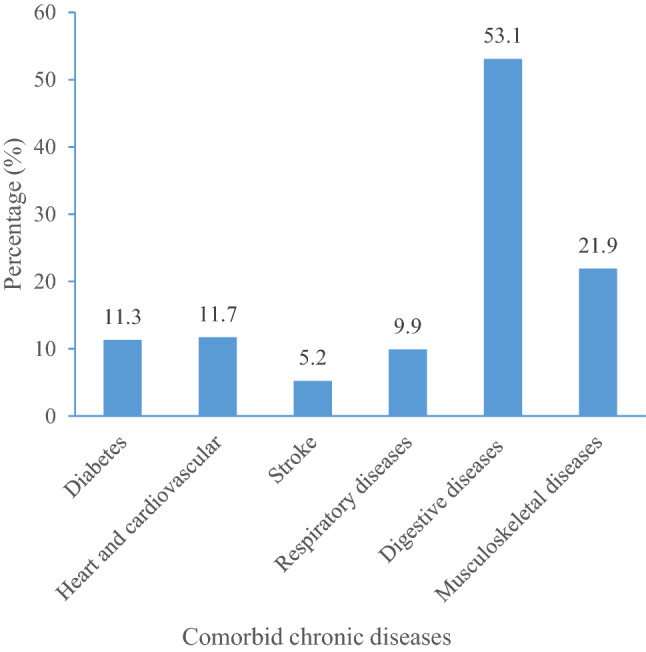
Percentage of BCSs with comorbid chronic diseases.

Figure 1 presents the number and percentage of comorbidities among BCSs. In this study, six types of chronic diseases were investigated: diabetes mellitus (11.3%), heart and cardiovascular diseases (11.7%), stroke (5.2%), respiratory diseases (9.9%), digestive diseases (53.1%), and musculoskeletal diseases (21.9%), were found among the participants. 67.8% of the participants had at least one comorbidity, of which 12.1% of the participants had three or more comorbidities. The average stigma score of all the participants was 1.62 points (SD = 0.59 points). The results from the multiple linear regression models are presented in Table 2. Models 3, 5, 6, and 7 were statistically significant and explained 3.1%, 3.0%, 3.1% and 3.6% of stigma among the participants, respectively. After controlling for sociodemographic factors and other health conditions, participants with stroke, digestive diseases, or musculoskeletal diseases had significantly higher stigma scores than those without these disorders. Compared with participants without comorbidities, participants with 1–2 chronic diseases and participants with 3 or more comorbidities had significantly higher stigma scores.

**Table 2:** Associations between comorbid chronic diseases and stigma in BCSs.

Model	Characteristics	Unstandardized coefficient (SE)	t	F	R^2^
Model 1	Diabetes (11.3%)	0.006 (0.023)	0.252	1.381	0.024
Model 2	Heart and cardiovascular (11.7%)	− 0.034 (0.023)	− 1.454	1.513	0.026
Model 3	Stroke (5.2%)	− 0.083 (0.033)	− 2.535*	1.789*	0.031
Model 4	Respiratory diseases (9.9%)	− 0.036 (0.025)	− 1.436	1.509	0.026
Model 5	Digestive diseases (53.1%)	− 0.035 (0.015)	− 2.389*	1.743*	0.030
Model 6	Musculoskeletal diseases (21.9%)	− 0.046 (0.018)	− 2.535*	1.789*	0.031
Model 7	Number of comorbidities			1.972*	0.036
	0 (32.2%)				
	1–2 (55.7%)	− 0.038 (0.016)	− 2.330*		
	≥ 3 (12.1%)	− 0.081 (0.025)	− 3.182**		

SE, standard error. *p < 0.05; **p < 0.01. In all these models, the dependent variable stigma was inverted transformed. And the following confounding factors were considered in each model: age, BMI and time since diagnosis as continuous variables, marital status, surgery, radiotherapy, chemotherapy, endocrine drug therapy, hysterectomy, recurrence and metastasis as dichotomous variables, education level and household per capital income as multi-categorical variables were converted into dummy variables before being included in the regression models. In model 7, the independent variable number of comorbidities was also converted into dummy variable (set dummy variables c1, c2; when number of comorbidities = 0, c1 = 0 and c2 = 0; when number of comorbidities = 1–2, c1 = 1 and c2 = 0; when number of comorbidities ≥ 3, c1 = 0 and c2 = 1). In all these models, all the variation inflation factor (VIF) values were below 10.

## Discussion

This study evaluated the association between comorbidities and stigma in BSCs. The results showed that, after controlling for confounding factors, participants with stroke, digestive diseases or musculoskeletal diseases had significantly higher stigma scores than those without these diseases. Also, participants with more comorbidities had higher stigma scores.

In this study, there were no statistically significant associations between social demographic factors and stigma among the breast cancer survivors, which differs from previous studies, showing that stigma is influenced by many socio-demographic (such as age, education level, marital status), clinical and psychological factors^46^. Although our study did not find meaningful correlations, it showed similar tendencies on some variables. Therefore, these demographic characteristics remained factors that couldn’t be ignored in the study of stigma and should be given attention. We controlled for these factors as confounders when exploring the association between comorbidities and stigma.

In addition to sociodemographic factors, treatment modalities were also found to be unrelated to stigma among BCSs. However, participants who received surgery, radiotherapy, chemotherapy or endocrine drugs showed a tendency to have higher levels of stigma. Breast cancer treatment leads to physical disability and physical changes^5,10^, which damage the self-esteem of patients and cause strong feelings of shame as they perceive themselves as losing their femininity^47,48^. However, some studies claimed that the stigma was not related to the specific treatment and that patients felt the same stigma whether they underwent mastectomy or breast-conserving treatment^49^. In this study, participants with metastasis had a significantly higher level of stigma. For BCSs with metastases, the disease is more severe and the treatment may be more complicated, which makes them extremely psychologically stressed and more likely to perceive discrimination. Therefore, they are more prone to be victims of stigma^20^.

In this study, several common chronic diseases, such as stroke, digestive diseases and musculoskeletal diseases, were related to the stigma of BCSs. These common chronic diseases exacerbate physical or financial burdens, which may enhance the overall perceived stigma of BCSs and further increase internalized stigma. Stroke patients usually have a limitation of activities caused by physical impairment, and are at increased risk of emotional processing disorders due to reduced social interaction and participation, resulting in increased stigma^30,50^. Stigma is also an important health issue in gastrointestinal diseases, which is related to the negative impression of society on such diseases and the serious psychological burden of patients. Patients with such diseases often feel embarrassed and reluctant to seek treatment and nursing, which brings more serious health problems^51^. Musculoskeletal diseases bring about reduced physical activity, weakness, decreased well-being and loss of independence, and seriously limit the ability of patients to change their lifestyle, thereby affecting the social network of patients and bringing stigma^52,53^.

The average score of stigma in this study was 1.62 points (± 0.59 points), which was higher than that in Iran (1.47 points ± 0.19 points)^38^. The difference may be related to the different cultural backgrounds, perceptions and acceptance of the disease in different countries. Rooted in the values of interdependence, Chinese culture emphasizes the importance of maintaining one's *face* and social status^54^. Previous studies have shown that some traditional Chinese beliefs include the idea that disease is the result of bad karma, or the punishment for prior misdeeds conducted by the individuals or their ancestors^55–57^. Therefore, the diagnosis of breast cancer or chronic diseases has the potential to undermine a person's perceived social status, which may lead to shame and self-blame, bringing with it a severe sense of stigma^58^. Stigma has been reported in AIDS patients^59^. The coexistence of disease, poverty and drug abuse will increase stigma^59^. Among BCSs, the economic burden caused by disease treatment, nursing and loss of work, the serious decline of physical function and the dependence on daily nursing may facilitate thoughts of uselessness. Survivors may define themselves as the burden of the whole family, and produce negative self-perception of themselves, which is the source of stigma^9^. Moreover, patients with both cancer and comorbidities may have a more severe decline in physical function, require more nursing and care, and therefore may have a greater degree of stigma. In this study, breast cancer and comorbidities can also lead to an overlay of stigma. And the higher the number of comorbidities, the more severe the stigma. Therefore, the issue of stigma in BCSs with comorbidities needs attention.

R-squared values in this study were small to some extent. Usually in the prediction model, the larger the R-squared value, the better the regression model fits the observations^60^. Fortunately, despite the low R-squared value, if the independent variables are statistically significant, important conclusions can still be drawn about the relationships between the variables^60^. In this study, regression models were used to explore the association between types and numbers of comorbidities and stigma among BCSs. Stigma is a very complex variable related to multiple factors in individual-related, disease-related, social contact, and support network dimensions^61,62^. However, due to various constraints, the independent variables included in this study were limited and the explanation of stigma was also limited. Nevertheless, the association between the types and numbers of comorbidities and stigma among BCSs is undeniable. Thus, this study calls for attention to the population of BCSs with comorbidities in psychological interventions such as stigma.

There are some limitations in this study. Firstly, respondents could have responded in a positive manner. Thus, stigma was not a guaranteed presence for all respondents and there was no way of determining the experience of stigma for each respondent prior to administering the questionnaires. Secondly, the sample might not be representative of an entire population, therefore findings were limited to this sample of respondents. Thirdly, the study collected online questionnaires through WeChat, a Chinese social networking platform. This survey method could lead to bias in content understanding as the investigator and participants couldn’t communicate face-to-face. However, in the context of COVID-19 pandemic, such online investigation had its unique advantages in terms of organization and implementation^36^. Besides, online surveys faced the risk of poor questionnaire quality, as participants could respond without reading the questions or submit questionnaires repeatedly^36,39^. To avoid these situations, we made some settings at the technical level, including limiting people using the same device or the same WeChat account to fill out the questionnaire only once and setting multiple logic test questions^36,39^. Finally, this study was a cross-sectional study, which had the disadvantage of insufficient demonstration of causality among research variables. Further research is still needed to clarify the causal associations of the association among similarly variables or factors.

## Conclusions

In conclusion, the study indicated that the stigma of BCSs with certain chronic diseases (stroke, digestive diseases or musculoskeletal diseases) was significantly higher than that of those who are not suffering from these diseases, and the stigma of BCSs was higher among those with more comorbidities. BCSs with comorbidities may be a priority population at risk for stigma and need more attention in the future long-term care and psychological interventions for community BCSs.

## Data Availability

The datasets generated during and/or analyzed during the current study are available from the corresponding author on reasonable request.
